# Axial spondyloarthritis, psoriatic arthritis and systemic lupus erythematosus share common molecular features based on post-hoc analysis of serum biomarkers

**DOI:** 10.1007/s00296-025-06012-0

**Published:** 2025-10-30

**Authors:** Rikke Malmkvist, Anne-Christine Bay-Jensen, Dovile Sinkeviciute, Signe Holm Nielsen, Monica Toft Hannani, Peder Frederiksen, Morten Asser Karsdal, Sheng Gao, Warner Chen

**Affiliations:** 1 Department of Biomedical Sciences, Faculty of Health and Medical Sciences, University of Copenhagen, Copenhagen, Denmark; 2 Nordic Bioscience, Herlev, Denmark; 3 Immunology, Janssen Research & Development, LLC, Spring House, PA USA

**Keywords:** Axial spondyloarthritis, Psoriatic arthritis, Systemic lupus erythematosus, Biomarkers, Cluster analysis, Phenoendotype

## Abstract

Axial spondyloarthritis (axSpA), psoriatic arthritis (PsA), and systemic lupus erythematosus (SLE) are distinct rheumatological diseases with common molecular features. This study explored tissue-associated remodelling endotypes using blood-based biomarkers reflecting interstitial matrix and basement membrane remodelling. Baseline serum samples from radiographic axSpA (r-axSpA) (NCT02437162/NCT02438787) (*n *= 66), PsA (NCT03158285) (*n *= 267) and SLE (NCT02349061) (*n *= 97) studies were analysed alongside healthy donors. Biomarkers of collagen turnover (C1M, C2M, C3M, C4M, C6M, C10C, PRO-C1, PRO-C2, PRO-C3, PRO-C4, PRO-C6) were measured by immunoassays and compared using Kruskal-Wallis tests. K-means clustering (*k *= 3) of r-axSpA, PsA and SLE patients was applied to construct disease endotype clusters. Serum C1M, C3M, C4M, C6M, and PRO-C3 markers were elevated up to 4.2-fold (*p *<0.003) in r-axSpA, PsA and SLE patients versus healthy donors. Serum C10C marker was elevated up to 1.2-fold (*p *< 0.001) in PsA and SLE patients versus healthy donors. PsA and r-axSpA patients showed increased levels of serum PRO-C2, PRO-C4, and PRO-C6 up to 1.4-fold (*p *< 0.025) versus healthy donors. Three endotypes were identified: 1) hypertrophic (*n *= 171), with elevated C10C, low PRO-C4 and lower disease activity reflected by CRP, ASDAS (r-axSpA), PASI, DAS28 (PsA) and joint scores (SLE); 2) fibrolytic (*n *= 156), with high tissue degradation, increased PRO-C4 and severe inflammation with elevated disease activity; 3) fibrogenic (*n *= 93), with increased cartilage degradation (C2M), fibrosis (PRO-C3, PRO-C6) and moderate disease activity. Tissue-associated remodelling profiles highlight heterogeneity within each disease and common molecular features, revealing clinically relevant subgroups. Identifying biomarker endotypes may guide targeted treatments and inform drug repurposing across rheumatological diseases.

**Supplementary Information:**

The online version contains supplementary material available at 10.1007/s00296-025-06012-0.

## Introduction

Axial Spondyloarthritis (axSpA), psoriatic arthritis (PsA) and systemic lupus erythematosus (SLE) are distinct rheumatic diseases characterised by unique clinical manifestations, such as spinal inflammation and sacroiliitis in axSpA, peripheral joint and skin involvement in PsA, and systemic autoimmunity with multiorgan involvement in SLE. Despite their clinical differences, these conditions share underlying molecular mechanisms, including chronic inflammation, abnormal activation of innate immune pathways, and dysregulated type I interferon signalling. Central to their pathology is an imbalance between degradation (fibrolysis) and synthesis (fibrogenesis) of connective tissue in affected tissues. Despite these shared molecular underpinnings, the considerable heterogeneity exhibited by these diseases presents substantial challenges in both diagnosis and treatment strategies [1].

Rheumatic diseases are characterised by chronic inflammation and developing tissue damage, leading to structural and functional alterations in affected tissues. Conventional methods of determining disease activity such as clinical assessment, laboratory tests and imaging have limitations in fully assessing the tissue damage extent and accurately predicting disease progression [2, 3]. In contrast, soluble biomarkers reflect ongoing processes of inflammation, extracellular matrix turnover (ECM), and tissue remodelling, offering a deeper insight into disease pathogenesis and activity.

In rheumatology, soluble biomarkers hold potential for enhancing diagnostic accuracy, monitoring disease activity and assessing treatment responses [1]. Noteworthy examples of current biomarkers widely employed in standard clinical practice, including in evaluation of axSpA, PsA and SLE, include C-reactive protein (CRP), erythrocyte sedimentation rate (ESR) and assessment of seropositive status. For SLE, soluble biomarkers such as anti-dsDNA antibodies, complement components (C3 and C4), are part of the diagnostic process. Soluble collagen biomarkers are molecules identified in bodily fluids, including blood, urine and synovial fluid, that serve as indicators of collagen metabolism and thereby reflects ongoing pathological processes.

Collagen is the predominant protein in the ECM of connective tissues, providing structural support and mechanical strength to various organs such as bone, cartilage and skin. Collagen degradation is essential for tissue renewal, remodelling and wound healing. Pathological conditions can trigger excessive collagen fibrolysis or fibrogenesis, as seen in therapeutic areas such as fibrosis, arthritis and cancer [4]. A prime example is the measurement of degradation products of type I collagen, such as C-telopeptide of type I collagen (CTX-I), which reflect bone resorption in osteoporosis [5]. In contrast, measuring degradation products of type II collagen, such as C-telopeptide of type II collagen (CTX-II), allows assessment of cartilage degradation in diseases such as osteoarthritis (OA) and rheumatoid arthritis (RA) [6, 7]. These biomarkers have demonstrated prognostic value in predicting disease progression and treatment responses [8], emphasising the important role of tissue remodelling and its corresponding biomarkers in disease pathogenesis.

Other relevant biomarkers include PRO-C3 (PIIINP) and C3M, which indicate the formation and degradation of type III collagen in tissues such as the synovium, liver, intestine and lungs (Table 1). Research has shown that collagen turnover is altered in various rheumatic diseases such as RA, PsA, systemic sclerosis (SSc) and axSpA, while SLE presents elevated basement membrane biomarkers (Table 1) [9–12]. Studies show that efficacious therapies, such as TNF and anti-IL6 receptor blockers, as well as Jak inhibitors, can alter the release of these collagen biomarkers in RA and axSpA. In contrast, therapies with limited effect, such as a Syk inhibitor, do not show the same effect on the biomarkers in RA [13–16]. These observations indicate the potential of collagen biomarkers to provide insights into the mechanism of action of these drugs at the tissue level.

**Table 1:** Overview of tissue-remodelling biomarkers investigated in this study

Biomarker	Analyte	Description
C1M	Type I α1 collagen fragments generated by MMP-1, -2, -8, -13 and -14 [17]	Reflects connective tissue destruction. Elevated across multiple disease in the inflammatory space including PsA, axSpA and RA [18–20]
C2M	Type II collagen fragments generated by multiple MMPs [21].	Reflects cartilage degradation. Released from cartilage in response to proinflammatory cytokines such as TNF [21]
C3M	Type III collagen fragments generated mainly by MMP-9 [22]	Reflects interstitial matrix degradation. Elevated across multiple disease in the fibroinflammatory space including PsA, axSpA and RA [10, 18, 19]
C4M	Type IV collagen fragments generated mainly by MMP-2 and -9 [22]	Reflects basement membrane degradation. Elevated across multiple disease in inflammatory space including RA and SSc [9, 18]
C6M	Type VI collagen fragments generated mainly by MMP-2 and -9 [23]	Reflects interstitial matrix degradation. Elevated across multiple disease in fibroinflammatory space including axSpA and SSc [19, 24]
C10C	Type X collagen fragments generated by cathepsin K (NC1 domain) [25]	Reflects endochondral bone modelling/remodelling. Produced by hypertrophic chondrocytes. Elevated in OA, PsA and axSpA [25, 26]
PRO-C1	The N-terminal propeptide of type I collagen [27]	Reflects both bone modelling/remodelling and fibrosis. Lowered levels observed in SSc [24]
PRO-C2	The N-terminal propeptide of type II collagen (PIIBNP) [28]	Reflects cartilage formation. Lower in OA and elevated in axSpA and PsA [26, 29]
PRO-C3	The N-terminal propeptide of type III collagen (PIIINP) [30]	Reflects connective fibrogenesis. Elevated in SSc and PsA [10, 24]
PRO-C4	The type VI collagen 7S domain [31]	Reflects basement membrane remodelling. Elevated in SSc [24]
PRO-C6	The C-terminus cleavage of type VI collagen (C5 domain, endotrophin) [32]	Reflects interstitial matrix formation. Elevated in SSc and PsA [10, 24]
VICM	Citrullinated and MMP degraded fragment of vimentin [33]	Reflects mononuclear cell activation, mainly macrophages. Elevated in RA and axSpA [18, 34]

References are selected as examples of the signal observed for disease compared to healthy controls in the fibroinflammatory space with focus on rheumatology

*axSpA* Axial Spondyloarthritis, *MMP* Matrix metalloproteinases, *OA* Osteoarthritis, *PsA* Psoriatic Arthritis, *RA* Rheumatoid Arthritis, *SSc* Systemic Sclerosis, *TNF* Tumour necrosis factor

Understanding how collagen biomarkers are regulated, along with identifying common biomarker profiles across different diseases, could lead to new tools for drug development and advance precision medicine for improved patient management [1, 35]. However, while individual biomarkers of inflammation and tissue remodelling have been studied within single rheumatic diseases, there is a lack of research comparing tissue-associated remodelling profiles across multiple conditions. In particular, no studies to date have investigated whether shared or disease-specific collagen-related biomarker profiles exist among radiographic axial spondyloarthritis (r-axSpA), PsA, and SLE. Addressing this gap could improve our understanding of common and distinct pathogenic pathways and support development of endotype-based patient stratification.

This exploratory study therefore aimed to assess the extent of tissue damage and fibrosis-related blood biomarkers among individuals with r-axSpA, PsA and SLE. The primary goal was to discern shared and distinctive attributes that characterise tissue-associated remodelling endotypes across diseases. Biomarkers were chosen based on a hypothesis-driven strategy focusing on collagens present in the interstitial matrix and basement membrane, both recognised to undergo remodelling in the three conditions. Additionally, citrullinated vimentin (VICM), a known biomarker in RA, was included due to its relevance in inflammatory tissue remodelling [36].

## Methods

### Study population

These post hoc analyses utilised subsets of available baseline serum samples with adequate volume from consenting patients of the r-axSpA (NCT02437162/NCT02438787) [37], PsA (NCT03158285) [38] and SLE (NCT02349061) [39] studies. Full methods and results of these studies have been reported previously. Briefly, the two r-axSpA phase 3 studies of ustekinumab (NCT02437162/NCT02438787) enrolled patients with active r-axSpA despite previous therapy. Patients had inadequate response to or were intolerant of nonsteroidal anti-inflammatory drugs and were TNFi-naïve (NCT02437162) or had inadequate response or intolerance to one TNFi (NCT02438787). The DISCOVER-2 (NCT03158285) phase 3 study enrolled biologic-naïve patients with active PsA despite standard therapies. The phase 2a study of ustekinumab (NCT02349061) enrolled patients who had a diagnosis of SLE at least three months before the first administration of study drug.

The four clinical studies were conducted in accordance with the Declaration of Helsinki and Good Clinical Practice. The protocols were approved by the institutional review board or ethics committee at each site. All patients gave written informed consent.

Healthy donor samples were acquired from the two commercial sources Discovery Life Sciences (San Luis Obispo, CA, USA) and BioIVT (West Sussex, UK), and samples were collected according to the European Union General Data Protection Regulation and U.S. ethical regulations and privacy act. Healthy donors were matched on age, sex and race with the collective disease cohorts (Supplementary Table S1).

### Biomarker quantification

Serum samples were collected from healthy donors (*n *= 77), r-axSpA (*n *= 66), PsA (*n *= 267) and SLE (*n *= 97) for biomarker analysis. The blood-based extracellular matrix degradation and formation biomarkers were measured in all participants using following available assays: nordicC1M™, nordicC2M™, nordicC3M™, nordicC4M™, nordicC6M™, nordicC10C™, nordicPRO-C1™, nordicPRO-C2_HP™, nordicPRO-C3™, nordicPRO-C6™, nordicPRO-C4™ and nordicVICM™. A detailed description of the biomarkers can be found in Table 1. All biomarkers were assessed by competitive immunoassays using manual ELISAs, except PRO-C4 which was measured the immunodiagnostic systems robotic platform (IDS-i10; Immunodiagnostics Systems (IDS), Bolden, Tyne & Wear, UK), and previously validated to measure in human serum samples. Samples were re-run if the duplicate coefficient of variation was >15%. The inter- and intra-assay coefficients of variation were <15 and <10% respectively for all assays.

### Statistical analysis

Biomarker levels were compared across healthy donors, r-axSpA, PsA and SLE patients using Kruskal-Wallis tests with Dunn’s test applied post-hoc. Holm’s method was used to adjust *p* values for multiple testing. Before clustering, the biomarker levels were log-transformed and standardised by median centring and scaling by the median absolute deviation (MAD). Euclidian distance was used as similarity metric, and *K*-means clustering was performed. Since the population consists of three disease groups, the number of clusters (*k*) was set to three. Biomarker levels in each cluster were evaluated post-hoc with Mann-Whitney *U* tests in comparison to healthy donors. Clinical variables were compared across clusters using χ^2^-tests for categorical variables and the Kruskal-Wallis tests for continuous variables. Differences were considered statistically significant when *p *≤ 0.05. All analyses were performed using R Statistical Software (v4.4.1; R Core Team 2024).

## Results

### Patient demographics and characteristics

The study included a cohort of 507 participants, comprising healthy donors (*n *= 77) as well as individuals diagnosed with r-axSpA (*n *= 66), PsA (*n *= 267), or SLE (*n *= 97). Notably, all enrolled patients exhibited active disease at the baseline when the samples were collected (Table 2).

**Table 2:** Demographics and clinical characteristics of all patient groups included in this study

	Healthy (*n* = 77)	r-axSpA (*n* = 66)	PsA (*n* = 267)	SLE (*n *= 97)	*p* value
Age, years	45.0 (32.0, 54.0)	40.5 (30.2, 49.0)	46.0 (37.0, 54.0)	41.0 (32.0, 49.0)	0.002
Sex, female	39 (50.6%)	13 (19.7%)	122 (45.7%)	88 (90.7%)	< 0.001
BMI, kg/m^2^		24.4 (21.5, 27.6)	27.8 (24.7, 32.5)	27.1 (23.1, 32.4)	< 0.001
Race					
White	51 (66.2%)	47 (71.2%)	260 (97.4%)	66 (68.0%)	
Hispanic	14 (18.2%)	0 (0.0%)	0 (0.0%)	0 (0.0%)	
Black	8 (10.4%)	1 (1.5%)	0 (0.0%)	7 (7.2%)	
Asian	4 (5.2%)	17 (25.8%)	7 (2.6%)	14 (14.4%)	
Other	0 (0.0%)	1 (1.5%)	0 (0.0%)	10 (10.3%)	
Disease duration, years		7.0 (3.0, 12.0)	PsO: 13.0 (8.0, 23.0) PsA: 4.3 (2.0, 8.3)	7.5 (3.4, 13.5)	
Disease Biomarkers		CRP: 1.7 (0.9, 2.7) mg/dL HLA-B27: 63 (95.5%)	CRP: 1.5 (0.7, 2.7) mg/dL	Anti-dsDNA: 52.6 (12.3, 158.8) IU/mL C3: 1.0 (0.7, 1.2) g/L C4: 0.1 (0.1, 0.2) g/L	
Disease activity and severity		ASDAS: 4.4 (3.9, 4.9) BASDAI: 7.4 (5.9, 8.4)	PASI: 6.0 (2.6, 13.0) IGA: 3.0 (2.0, 3.0) DAS28: 5.3 (4.7, 6.0) BSA: 10.0 (4.0, 23.0) SJC66: 11.0 (8.0, 16.0) TJC68: 19.0 (12.0, 29.0) vdH-S: 16.5 (7.5, 37.2) HAQ: 1.4 (1.0, 1.8)	SLEDAI: 10.0 (8.0, 12.0) PGA: 5.0 (3.8, 5.9) CLASI: 5.0 (2.0, 10.0) SJC62: 5.0 (3.0, 8.0) TJC64: 8.0 (4.0, 15.0) AJC62: 5.0 (3.0, 8.0) 15 (15.5%) had lupus nephritis	

Data are presented as median (interquartile range) or n (%). Where applicable; differences were assessed by Kruskal-Wallis tests or χ^2^**-**tests with Holm-adjusted *p* values

*AJC62* active joint count in 62 joints, *ASDAS* ankylosing spondylitis disease activity score, *BASDAI* bath ankylosing spondylitis disease activity index, *BMI* body mass index, *BSA* body surface area, *C3* complement component 3, *C4* complement component 4, *CLASI* cutaneous lupus erythematosus disease area and severity index, *CRP* c-reactive protein, *DAS28* disease activity score in 28 joints, *HAQ* health assessment questionnaire, *HLA* human leukocyte antigen, *IGA* investigator’s global assessment, *PASI* psoriasis area severity index, *PGA* physician global assessment, *PsA* psoriatic arthritis, *PsO* psoriasis, *r-axSpA* radiographic axial spondyloarthritis, *SJC66/62* swollen joint count in 66/62 joints, *SLE* systemic lupus erythematosus, *SLEDAI* systemic lupus erythematosus disease activity index, *TJC68/64* tender joint count in 68/64 joints, *vdH-S* van der Heijde-Sharp

### Elevated levels of tissue degradation markers

**Fig. 1:**
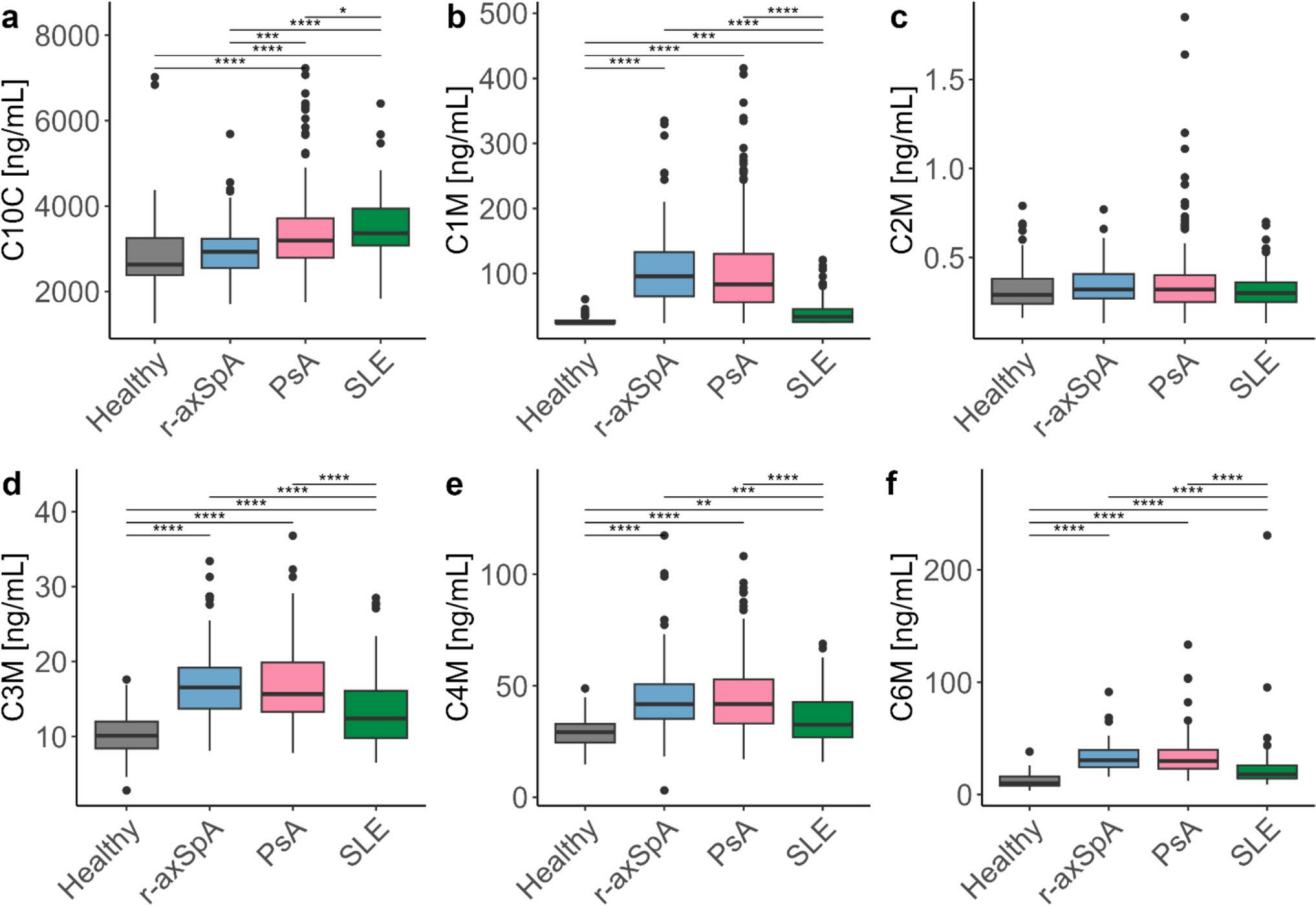
Comparing collagen degradation biomarkers across healthy and disease cohorts. **a** C10C, **b** C1M, **c** C2M, **d** C3M, **e** C4M, **f** C6M. Data are shown as boxplots with medians and interquartile ranges. Differences were assessed by Kruskal-Wallis test with Dunn’s test applied post-hoc and Holm adjusted *p* values where *p ≤ 0.05, **p ≤ 0.01, ***p ≤ 0.001, **** *p* ≤ 0.0001

PsA and SLE patients had elevated C10C levels (median [interquartile range]: 3.19 [2.79, 3.71] µg/mL, 3.37 [3.08, 3.94] µg/mL, respectively) compared to healthy donors (2.63 [2.38, 3.25]) (*p*<0.001 for both) (Fig. 1a). Patients with r-axSpA had elevated levels of type I (C1M) (95.8 [65.0, 133.1] ng/mL), type III (C3M) (16.5 [13.7, 19.2] ng/mL), type IV (C4M) (41.8 [35.2, 50.7] ng/mL) and type VI (C6M) (30.6 [24.3, 39.7] ng/mL) collagen degradation compared to healthy donors (23.6 [23.6, 27.9] ng/mL, 10.1 [8.4, 12.0] ng/mL, 29.2 [24.6, 32.9] ng/mL, 10.1 [7.9, 16.1] ng/mL, respectively) (*p*<0.001 for all) (Fig. 1b, d–f). PsA patients also had increased levels of type I (83.3 [56.2, 130.3] ng/mL), type III (15.6 [13.3, 19.9] ng/mL), type IV (41.8 [33.1, 52.9] ng/mL) and type VI (29.9 [23.1, 39.8] ng/mL) collagen degradation compared to healthy donors (*p*<0.001 for all). SLE patients had increased type I (33.6 [25.8, 45.5], ng/mL), type III (12.4 [9.8, 16.1] ng/mL), type IV (32.6 [26.9, 42.7] ng/mL) and type VI (18.0 [14.3, 25.9] ng/mL) collagen degradation compared to healthy donors (*p*<0.001 for all, except type IV *p*=0.003). Levels of these markers were similar between r-axSpA and PsA patients, where SLE patients generally had relatively low levels. Degradation marker of type II (C2M) collagen did not show any difference between any of the groups (Fig. 1c).

### Alterations in tissue formation and turnover markers

**Fig. 2:**
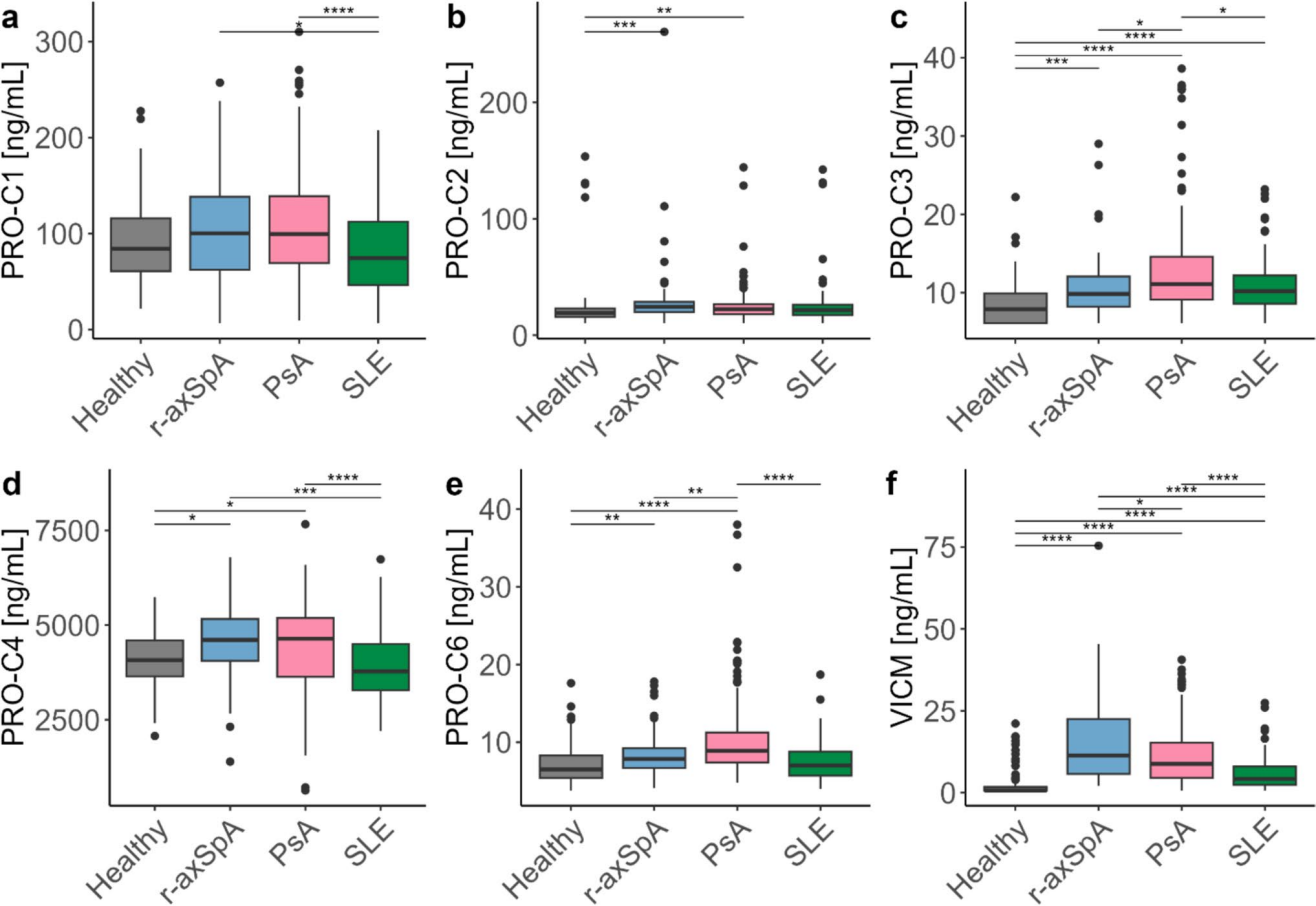
Comparing collagen formation/turnover and inflammation biomarkers across healthy and disease cohorts. **a** PRO-C1, **b** PRO-C2, **c** PRO-C3, **d** PRO-C4, **e** PRO-C6, **f** VICM. Data are shown as boxplots with medians and interquartile ranges. Differences were assessed by Kruskal-Wallis test with Dunn’s test applied post-hoc and Holm adjusted *p* values

None of the patient groups had different levels of PRO-C1 compared to healthy donors (Fig. 2a), however SLE patients (74.5 [46.4, 112.5] ng/mL) did have lower levels compared to r-axSpA (100.3 [62.5, 138.4] ng/mL, *p*=0.05) and PsA patients (99.6 [69.4, 139.1] ng/mL, *p*<0.001). Patients with r-axSpA had increased levels of PRO-C2 (24.4 [19.9, 28.7] ng/mL), PRO-C3 (9.9 [8.2, 12.1] ng/mL), PRO-C4 (4.60 [4.05, 5.16] ng/mL) and PRO-C6 (7.8 [6.7, 9.2] ng/mL) compared to healthy donors (19.2 [15.6, 23.0] ng/mL, 7.9 [6.1, 9.9] ng/mL, 4.07 [3.65, 4.60] ng/mL, 6.5 [5.4, 8.3] ng/mL, respectively) (*p*<0.001, *p*<0.001, *p*=0.025, *p*=0.008, respectively) (Fig. 2b–e). PsA patients also had elevated levels of PRO-C2 (22.3 [18.0, 26.6] ng/mL), PRO-C3 (11.1 [9.1, 14.6] ng/mL), PRO-C4 (4.64 [3.63, 5.19] ng/mL) and PRO-C6 (8.9 [7.4, 11.3] ng/mL) compared to healthy donors (*p*=0.006, *p*<0.001, *p*=0.025, *p*<0.001, respectively). SLE patients showed increased levels of PRO-C3 (10.2 [8.6, 12.2] ng/mL) compared to healthy donors (*p*<0.001), whereas PRO-C2, PRO-C4 and PRO-C6 did not show elevated levels.

The inflammatory marker, VICM, was elevated across diseases groups compared to healthy donors (0.7 [0.7, 1.8] ng/mL) with r-axSpA (11.3 [5.7, 22.5] ng/mL, *p*<0.001) and PsA (8.8 [4.5, 15.3] ng/mL, *p*<0.001) being more elevated than SLE (4.2 [2.4, 8.0] ng/mL, *p*<0.001) (Fig. 2f).

### Biomarker clustering and profile analysis

**Fig. 3:**
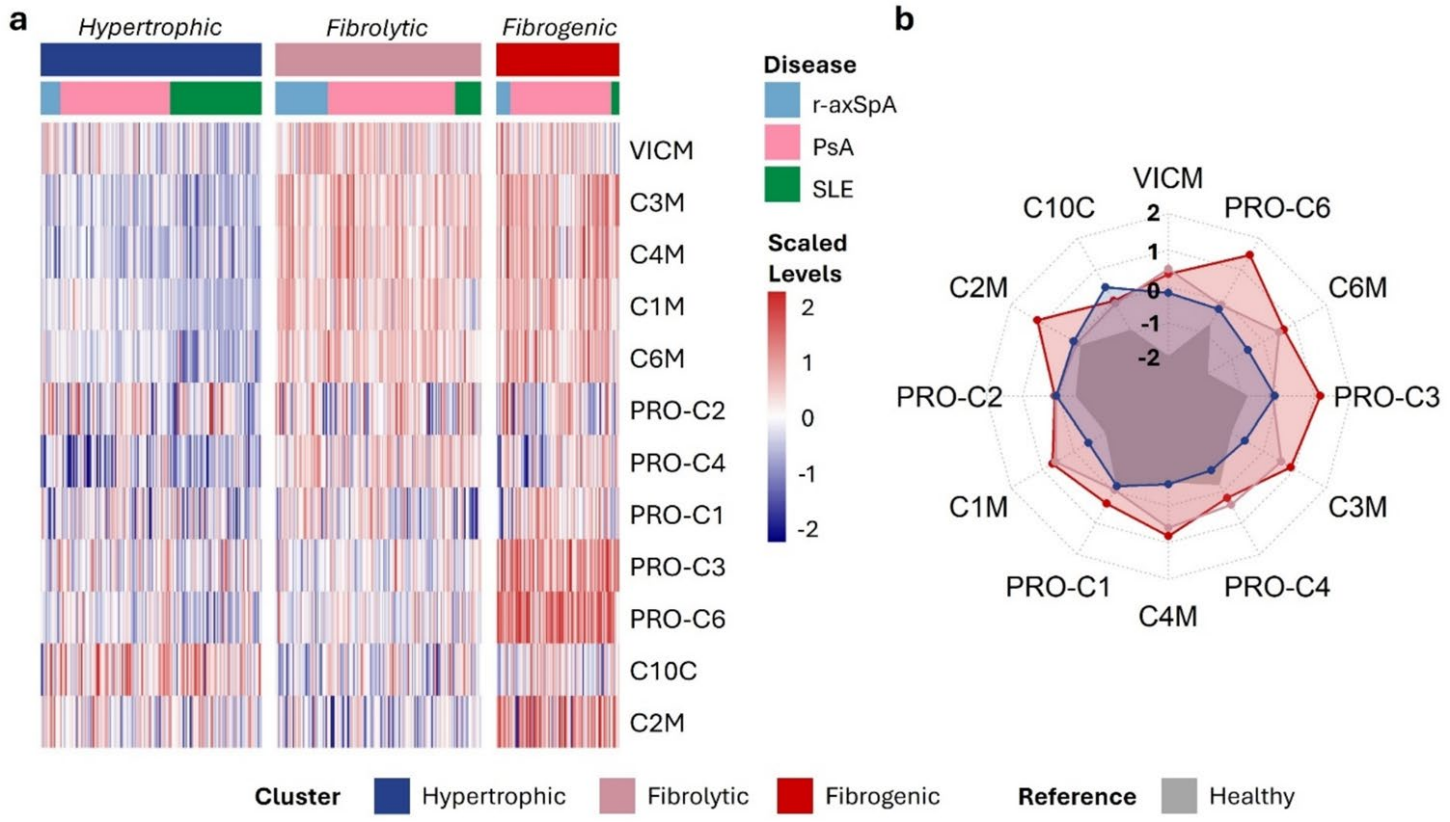
**a** Heatmap of standardised tissue-associated remodelling biomarker levels (rows) across individuals (columns) represented in the three constructed clusters from the *K*-means clustering analysis across the PsA, r-axSpA and SLE disease groups. **b** Radar plots of the median of standardised biomarker levels within each cluster. The grey area represents the profile of the healthy controls, not included in the clustering analysis, as a reference point

To discern whether the three disease groups shared underlying tissue-remodelling features or were unique from each other, the tissue-turnover biomarkers from the three disease cohorts were combined, and *K*-means clustering was applied constructing three clusters (*k*=3). This revealed patterns of similarity in the biomarker profiles across the diseases (Fig. 3), thereby providing insights into potential underlying relationships and shared characteristics within the studied populations despite the different diagnoses.

A comprehensive overview of the biomarker levels between the three constructed clusters as well as the healthy controls for comparison are presented in supplementary Table S2.

Cluster 1 (*n *= 171) was primarily driven by higher levels of C10C. As C10C is a marker indicative of hypertrophic chondrocytes (Table 1), this cluster was termed *hypertrophic cluster*. Cluster 2 (*n *= 156) was mainly driven by elevated levels of C1M, C3M, C4M and C6M, indicative of fibrolytic activity and the cluster was termed the *fibrolytic cluster*. Cluster 3 (*n *= 93) was driven by increased levels of PRO-C3 and PRO-C6, indicative of fibrogenesis and the cluster was termed the *fibrogenic cluster*.

Besides the higher levels of C10C, the hypertrophic cluster showed decreased levels of PRO-C4 compared to the healthy controls (post-hoc Mann Whitney *U* test, *p*<0.001). In addition to the elevated levels of the fibrolytic markers, the fibrolytic cluster had the highest levels of PRO-C4 out of all cluster and they were significantly elevated compared to healthy controls (*p*<0.001). The fibrogenic cluster also showed that the levels of C1M, C3M, C4M and C6M levels were significantly increased compared to the healthy donors (*p*<0.001). As the only cluster, the fibrogenic cluster showed significantly increased C2M levels compared to healthy donors (*p*<0.001). VICM levels were significantly elevated in all three clusters compared to healthy donors (*p*<0.001).

### Clinical features of the clusters

The hypertrophic cluster was the largest of the three clusters comprised of 86 PsA (50.3%), 70 SLE (40.9%) and 15 r-axSpA patients (8.8%). The fibrolytic cluster was the second largest cluster and contained 96 PsA (61.5%), 20 SLE (12.8%) and 40 r-axSpA patients (25.6%). The fibrogenic cluster was the smallest and consisted of 76 PsA (81.7%), 6 SLE (6.5%) and 11 r-axSpA patients (11.8%). The clinical characteristics and demographics of each disease group across the three clusters are detailed in Table 3.

**Table 3:** Clinical characteristics and demographics associated with each of the three tissue-associated remodelling clusters defined in the study

		Hypertrophic (*n = *171)	Fibrolytic (*n = *156)	Fibrogenic (*n = *93)	*p* value
r-axSpA	N (%)	15 (22.7%)	40 (60.6%)	11 (16.7%)	
	Age, years	40.0 (28.0, 41.5)	40.5 (30.0, 49.0)	52.0 (38.0, 60.0)	0.097
	Females, n (%)	3 (20.0%)	5 (12.5%)	5 (45.5%)	0.103
	BMI, kg/m^2^	22.7 (21.6, 27.1)	25.2 (22.1, 28.4)	24.4 (19.0, 26.5)	0.962
	r-axSpA duration, years	7.0 (1.8, 10.5)	6.2 (3.1, 11.1)	10.0 (5.1, 13.8)	1.000
	HLA-B27	15 (100.0%)	39 (97.5%)	9 (81.8%)	0.103
	CRP, mg/dL	0.6 (0.4, 0.9)	1.8 (1.4, 3.2)	1.5 (1.0, 5.8)	**<0.001**
	ASDAS	3.7 (3.4, 4.0)	4.7 (4.2, 4.9)	4.3 (3.6, 5.5)	**0.001**
	BASDAI	7.3 (5.9, 8.0)	7.4 (6.3, 8.3)	8.2 (5.2, 9.2)	1.000
PsA	N (%)	86 (33.3%)	96 (37.2%)	76 (29.5%)	
	Age, years	48.5 (39.2, 55.0)	43.0 (34.0, 52.0)	47.5 (38.8, 56.0)	0.072
	Females, n (%)	52 (60.5%)	38 (39.6%)	27 (35.5%)	**0.002**
	BMI, kg/m^2^	29.1 (24.8, 32.7)	26.7 (24.3, 31.2)	27.9 (25.0, 33.3)	0.798
	PsO duration, years	4.2 (2.1, 8.4)	4.2 (1.7, 8.3)	4.0 (1.8, 8.0)	0.798
	PsA duration, years	13.0 (8.1, 25.0)	14.0 (9.9, 22.0)	11.0 (6.0, 20.2)	0.899
	CRP, mg/dL	0.7 (0.4, 1.1)	2.2 (1.3, 3.3)	2.5 (1.2, 3.9)	**<0.001**
	PASI	4.0 (1.4, 8.3)	8.0 (4.1, 15.7)	5.4 (2.6, 15.1)	**0.001**
	IGA score	2.0 (2.0, 3.0)	3.0 (2.0, 3.0)	3.0 (2.0, 3.0)	0.072
	DAS28	4.9 (4.3, 5.5)	5.4 (4.9, 6.1)	5.6 (4.9, 6.3)	**<0.001**
	BSA (PsO)	6.0 (2.2, 11.0)	11.5 (5.0, 30.0)	12.5 (4.0, 29.5)	**0.003**
	SJC66	9.1 (7.0, 13.0)	11.0 (8.0, 15.1)	12.0 (8.0, 19.0)	0.161
	TJC68	19.0 (11.0, 25.0)	18.5 (12.8, 30.0)	20.0 (13.0, 31.0)	0.798
	vdH-S	10.2 (4.5, 27.1)	22.5 (8.0, 47.5)	21.2 (11.9, 45.1)	**0.004**
	HAQ	1.4 (0.9, 1.8)	1.4 (1.0, 1.8)	1.4 (1.1, 1.9)	0.657
SLE	N (%)	70 (72.9%)	20 (20.8%)	6 (6.3%)	
	Age, years	40.0 (31.2, 48.8)	42.0 (32.8, 47.0)	40.0 (34.8, 53.5)	1.000
	Females, n (%)	63 (90.0%)	19 (95.0%)	5 (83.3%)	1.000
	BMI, kg/m^2^	26.5 (22.1, 30.9)	27.1 (25.3, 33.3)	30.0 (27.2, 33.1)	1.000
	SLE duration, years	8.4 (4.4, 15.0)	4.8 (1.5, 12.3)	4.8 (1.6, 8.1)	1.000
	Anti-dsDNA, IU/mL	66.2 (12.3, 151.7)	21.0 (12.3, 359.5)	23.0 (12.3, 130.1)	1.000
	C3, g/L	0.9 (0.7, 1.1)	1.1 (0.9, 1.4)	1.1 (0.8, 1.3)	0.355
	C4, g/L	0.1 (0.1, 0.2)	0.2 (0.1, 0.3)	0.1 (0.1, 0.2)	0.440
	SLEDAI	10.0 (8.0, 12.0)	10.0 (8.0, 12.0)	10.0 (8.5, 10.8)	1.000
	PGA score	5.2 (3.4, 6.1)	4.8 (3.7, 5.2)	4.8 (4.4, 5.2)	1.000
	CLASI	6.0 (3.0, 12.0)	4.0 (2.0, 7.8)	2.0 (2.0, 2.0)	0.600
	SJC62	5.0 (2.6, 7.0)	6.5 (3.0, 11.2)	5.8 (4.4, 12.8)	1.000
	TJC64	7.0 (4.0, 13.8)	12.5 (5.9, 18.2)	8.2 (6.5, 13.4)	1.000
	AJC62	4.8 (3.0, 7.4)	6.5 (3.0, 9.6)	5.8 (4.4, 12.8)	1.000
	Lupus Nephritis, n (%)	11 (15.7%)	3 (15.0%)	1 (16.7%)	1.000

Data are presented as median (interquartile range) or n (%). Differences were assessed by Kruskal-Wallis tests or chi squared tests with Holm adjusted *p* values with significant values highlighted in bold

*AJC62* active joint count in 62 joints, *ASDAS* ankylosing spondylitis disease activity score, *BASDAI* bath ankylosing spondylitis disease activity index, *BMI* body mass index, *BSA* body surface area, *C3* complement component 3, *C4* complement component 4, *CLASI* cutaneous lupus erythematosus disease area and severity index, *CRP* c-reactive protein, *DAS28* disease activity score in 28 joints, *HAQ* health assessment questionnaire, *HLA* human leukocyte antigen, *IGA* investigator’s global assessment, PASI psoriasis area severity index, *PGA* physician global assessment, *PsA* psoriatic arthritis, *PsO* psoriasis, *r-axSpA* radiographic axial spondylarthritis, *SJC66/62* swollen joint count in 66/62 joints, *SLE* systemic lupus erythematosus, *SLEDAI* systemic lupus erythematosus disease activity index, *TJC68/64* tender joint count in 68/64 joints, *vdH-S* van der Heijde-Sharp

The r-axSpA disease cohort showed difference in CRP levels between the clusters (*p*<0.001), where the fibrolytic cluster numerically had the highest levels, followed by the fibrogenic and hypertrophic. The same trends were seen in the AS disease activity score (ASDAS), whereas the bath AS disease activity index (BASDAI) was not notably different between the clusters, albeit slightly elevated in the fibrogenic cluster.

For the PsA disease cohort there was also a difference in CRP levels between the clusters (*p*<0.001), where the lowest levels were also seen in the hypertrophic compared to the other clusters consistent with what was seen for r-axSpA patients. Psoriasis area severity index (PASI) was numerically higher in the fibrolytic compared to the hypertrophic and fibrogenic cluster, whereas the disease activity score in 28 joints (DAS28) and the body surface area of psoriasis (BSA PsO) was highest in the fibrogenic and lowest in the hypertrophic cluster with the levels in the fibrolytic being closer to the fibrogenic cluster levels. Moreover, the van der Heijde Modified Sharp (vdH-S) score was elevated in the fibrogenic and fibrolytic clusters, respectively compared to the hypertrophic cluster.

No significant differences in the demographic and clinical characteristics were observed between the clusters for the SLE disease cohort. However, the joint scores (swollen joint count in 62 joints [SJC62], tender joint count in 64 joints [TJC64], active joint count in 62 joints [AJC62]) were numerically highest in fibrolytic cluster compared to the other clusters, although not statistically significantly.

## Discussion

The study aimed to discern both shared and distinct characteristics of tissue-associated remodelling endotypes in patients with r-axSpA, PsA and SLE compared to healthy controls. This was based on the initial hypothesis that using tissue-associated remodelling biomarkers to classify patients across different rheumatic disorders could highlight common pathways and potentially enable drug repurposing across diseases.

Our findings indicated elevated levels of tissue-associated remodelling biomarkers across the three diseases compared to healthy controls; however, the extent of elevations varied. Collagen degradation markers (C1M, C3M, C4M and C6M) were elevated across all disease groups compared to healthy controls, suggesting a common inflammatory-driven tissue turnover process. While r-axSpA and PsA showed similar levels of these markers, SLE patients generally exhibited lower levels, potentially indicating differences in joint and tissue involvement between diseases. The lack of difference in C2M levels across groups further supports the notion that type II collagen degradation, primarily associated with cartilage breakdown, may not be a dominant feature in these conditions.

Across all diseases groups tissue formation (PRO-C3) was increased, particularly in PsA, which may reflect heightened fibrotic or reparative responses. As PRO-C3 has been shown to be modulated in several skin diseases, this may be why PsA patients show increased levels compared to r-axSpA and SLE [40]. The elevation of PRO-C2, PRO-C4 and PRO-C6 in r-axSpA and PsA, but not in SLE, suggests that structural tissue remodelling in these conditions may differ, with SLE potentially having a less pronounced fibrotic component. Moreover, the inflammatory biomarker VICM was markedly elevated in r-axSpA and PsA, supporting the hypothesis of stronger inflammatory-driven tissue damage in these diseases compared to SLE. Additionally, it also confirms previous findings of VICM being upregulated and modulated in r-axSpA and PsA patients [14, 41].

Despite the discrepancies, there were no obvious indications of one disease measuring higher throughout the panel of biomarkers for degradation or formation; thus, an attempt to define endotypes across diseases based on biomarker levels was carried out. These comparative analyses within each disease group provide valuable insights into the demographic and clinical heterogeneity across different clusters, shedding light on potential associations between biomarker profiles and specific characteristics within these rheumatic conditions.

The clustering analysis provided additional insights into disease heterogeneity, identifying three clusters–hypertrophic, fibrolytic and fibrogenic–each reflecting different biological mechanisms. The hypertrophic cluster contained the majority of SLE patients and was characterised by high C10C, reflecting type X collagen turnover, which is primarily expressed in hypertrophic cells, such as those in the growth plate, where it plays a key role in bone development. To our knowledge, SLE and type X collagen has not previously been associated, however COL10A1 expression has been shown to regulated in various bone and cartilage pathologies [25]. Therefore, alterations to type X collagen turnover may indicate disrupted cartilage remodelling or dysregulation of specific tissue types. The hypertrophic cluster also showed low PRO-C4 levels, indicating that it is not only a subgroup with a distinct tissue remodelling pattern favouring degradation over repair in cartilage, but also in the basement membranes where collagen type IV is primarily found [24]. The fibrolytic cluster had the highest levels of multiple collagen degradation markers, aligning with a more aggressive inflammatory tissue destruction. It displayed a pronounced fibrolytic profile with a basement membrane turnover component reflected by high PRO-C4 levels. This indicated that this subgroup of patients can mainly be described by their tissue destruction profile. This is a profile which has been observed in RA patients; a strong interstitial matrix destruction with a low fibrotic component [18]. The fibrogenic cluster was associated with elevated PRO-C3 and PRO-C6 levels, indicating a distinct subgroup with increased matrix formation, potentially reflecting a reparative or fibrotic phenotype. PRO-C3 is a well-established marker for fibrosis staging, particularly in conditions such as liver fibrosis, and serves as a prognostic indicator in diseases such as interstitial lung fibrosis [30, 42]. Similarly, PRO-C6 is associated with fibrosis and is predictive of poor outcomes in heart failure patients [43]. The differential distribution of disease cohorts across clusters underscores the complex relations between inflammation and tissue remodelling, revealing potential endotypes that could inform treatment strategies.

From a clinical perspective, CRP and disease activity scores were significantly associated with the clustering, reinforcing the relevance of these biomarker profiles in disease severity stratification. SLE patients were largely absent from the fibrogenic cluster, suggesting a lower tendency for excessive matrix formation in this cohort, which may have implications for targeted therapeutic approaches.

This study has several limitations to consider. As an exploratory data analysis, it does not aim to define diagnostic biomarkers, and the collagen signals are not single disease specific. The healthy donors were sourced from a commercial vendor and differ in origin from the patient groups as well as being matched to the collective disease groups and may therefore not be ideal comparators for each disease. Finally, the post hoc design along with differences in disease activity, sample size, and sex distribution across groups present additional challenges that should be considered when interpreting the findings.

Despite these limitations, the study has notable strengths. It is, to our knowledge, the first study to investigate tissue-associated remodelling endotypes across multiple rheumatic diseases, r-axSpA, PsA, and SLE, using a unified panel of serological biomarkers. The multi-disease design allowed direct comparison of remodelling profiles across distinctly diagnosed conditions, revealing both share and disease-specific pathological processes. Stratifying patients into clusters based on biomarker endotypes emphasises heterogeneity within these diseases while also revealing biologically plausible subgroups associated with different levels of disease activity and remodelling processes.

From a drug development perspective, these findings hold promise for potential opportunities for therapeutic repurposing and the design of targeted treatment strategies guided by biomarker-defined endotypes. Examining each cluster individually may help provide deeper insights into the mechanistic drivers of the disease and inform precision therapeutic approaches for specific patient subgroups. While exploratory in nature, this study offers a valuable foundation for future research into tissue-associated remodelling-related pathogenic mechanisms and potential personalised therapeutic strategies for rheumatic diseases.

## Supplementary Information

Below is the link to the electronic supplementary material.

Supplementary file1 (DOCX 17 KB)

## Data Availability

The data-sharing policy of Janssen Pharmaceutical Companies of Johnson & Johnson is available at https://www.janssen.com/clinical-trials/transparency. As noted on this site, requests for access to the study data can be submitted through the Yale Open Data Access (YODA) Project site at http://yoda.yale.edu.
