# Supplementary material for: Axial spondyloarthritis, psoriatic arthritis and systemic lupus erythematosus share common molecular features based on post-hoc analysis of serum biomarkers
Source: Rheumatol Int. 2025 Oct 30;45(11):262. doi: 10.1007/s00296-025-06012-0 (PMC12575503; doi:10.1007/s00296-025-06012-0)
Supplement: Supplementary file 1 — Supplementary file1 (DOCX 17 KB) [file 296_2025_6012_MOESM1_ESM.docx]

SUPPLEMENTARY INFORMATION

Supplementary Table S1 Demographics and clinical characteristics of healthy controls and the patient cohorts collectively. Data are presented as median (interquartile range) or n (%). Where applicable; differences were assessed by Mann-Whitney *U* tests or χ^2^ tests with Holm adjusted *p* values.

|  | **Healthy**  (*n = 77*) | **Disease**  (*n = 430*) | ***p* value** |
| --- | --- | --- | --- |
| **Age, years** | 45.0 (32.0, 54.0) | 44.0 (34.0, 53.0) | 0.716 |
| **Sex, female** | 39 (50.6%) | 223 (51.9%) | 0.845 |
| **Race** |  |  | < 0.001 |
| **- White** | 51 (66.2%) | 373 (86.7%) |  |
| **- Hispanic** | 14 (18.2%) | 0 (0.0%) |  |
| **- Black** | 8 (10.4%) | 8 (1.9%) |  |
| **- Asian** | 4 (5.2%) | 38 (8.8%) |  |
| **- Other** | 0 (0.0%) | 11 (2.6%) |  |

Supplementary Table S2 Overview of the biomarker levels across the three constructed clusters including healthy controls for comparison. Data are presented as median (interquartile range) with unit *ng/mL*. Differences were assessed with Mann-Whitney *U* tests with holm adjusted *p*  values, where each cluster has been compared with the healthy controls.

| ***p* value** | **Fibrogenic** | <0.001 | <0.001 | 1 | <0.001 | <0.001 | <0.001 | 1 | 0.001 | <0.001 | <0.001 | <0.001 | <0.001 |
| --- | --- | --- | --- | --- | --- | --- | --- | --- | --- | --- | --- | --- | --- |
|  | **Fibrolytic** | 0.01 | <0.001 | 1 | <0.001 | 1 | <0.001 | 1 | 0.01 | <0.001 | 0.031 | 0.012 | <0.001 |
|  | **Hypertrophic** | <0.001 | <0.001 | <0.001 | <0.001 | <0.001 | <0.001 | 0.001 | 0.03 | <0.001 | 0.08 | <0.001 | <0.001 |
|  | **Healthy**  **(n = 74)** | 2575 (2383, 3146) | 23.6 (23.6, 27.7) | 0.28 (0.24, 0.38) | 10.1 (8.4, 12.1) | 29.2 (24.8, 33.6) | 10.1 (7.9, 15.8) | 83.0 (61.1, 115.8) | 18.9 (15.1, 22.8) | 7.7 (6.1, 9.7) | 4074 (3645, 4595) | 6.3 (5.3, 8.3) | 0.7 (0.7, 1.2) |
|  | **Fibrogenic**  **(n = 93)** | 3135 (2768, 3580) | 111.9 (78.1, 174.6) | 0.46 (0.34, 0.58) | 19.3 (16.0, 23.4) | 50.3 (40.0, 61.9) | 35.4 (27.6, 47.9) | 113.1 (84.1, 153.4) | 22.5 (17.0, 27.0) | 15.2 (12.9, 19.4) | 4561 (3728, 5171) | 12.8 (10.6, 16.0) | 9.2 (5.1, 16.3) |
|  | **Fibrolytic**  **(n = 156)** | 3080 (2634, 3347) | 101.9 (72.1, 139.2) | 0.30 (0.23, 0.34) | 17.4 (15.3, 20.4) | 46.4 (40.7, 53.8) | 32.7 (27.3, 41.2) | 89.5 (62.3, 123.9) | 22.3 (18.7, 27.0) | 9.8 (8.5, 11.2) | 4822 (4324, 5380) | 7.8 (6.8, 8.9) | 10.8 (5.6, 18.5) |
|  | **Hypertrophic**  **(n = 171)** | 3437 (3011, 4014) | 39.8 (28.4, 58.1) | 0.31 (0.25, 0.37) | 11.8 (10.4, 13.8) | 30.1 (26.1, 35.0) | 19.8 (16.4, 23.9) | 84.2 (56.8, 115.8) | 22.3 (17.7, 26.5) | 10.0 (8.4, 12.0) | 3659 (3124, 4498) | 7.5 (6.3, 8.8) | 5.1 (2.7, 9.4) |
|  |  | **C10C** | **C1M** | **C2M** | **C3M** | **C4M** | **C6M** | **PRO-C1** | **PRO-C2** | **PRO-C3** | **PRO-C4** | **PRO-C6** | **VICM** |
